# Epidemiology of transthyretin (ATTR) amyloidosis: a systematic literature review

**DOI:** 10.1186/s13023-025-03547-0

**Published:** 2025-01-16

**Authors:** Diego Delgado, Firas Dabbous, Nitin Shivappa, Faizan Mazhar, Eric Wittbrodt, Divya Shridharmurthy, Krister Järbrink

**Affiliations:** 1 Division of Cardiology and Transplant, UHN, Toronto, ON Canada; 2 Data Analytics - Real World Evidence, Evidera, Bethesda, MD USA; 3 Cardiovascular, Renal and Metabolism (CVRM) Evidence, BioPharmaceuticals Medical, AstraZeneca, Wilmington, DE USA; 4 Real-World Evidence, Data Analytics, Evidera, Stockholm, Sweden; 5 Cardiovascular, Renal and Metabolism (CVRM) Evidence, BioPharmaceuticals Medical, AstraZeneca, Gothenburg, Sweden

## Abstract

**Introduction:**

Significant advances in the treatment of transthyretin (ATTR) amyloidosis has led to an evolving understanding of the epidemiology of this condition. This systematic literature review (SLR) aims to synthesize current evidence on epidemiology and mortality outcomes in ATTR amyloidosis, addressing the need for a comprehensive understanding of its current global impact.

**Methods:**

An SLR of the literature from January 2018 to April 2023 was conducted using the Medline and Embase databases. The review followed the PRISMA guidelines. Studies evaluating populations with genotypes and phenotypes of ATTR amyloidosis (variant and wild-type cardiomyopathy, polyneuropathy, and mixed) were included. Observational studies, systematic reviews, and meta-analyses were eligible, while reports, commentaries, clinical trials, and non-ATTR amyloidosis studies were excluded. Extracted data included prevalence, incidence, and mortality rates.

**Results:**

Of the 1,458 studies identified, 113 met the inclusion criteria. Forty-nine studies reported on epidemiology, while 64 focused on mortality rates in cohorts of patients with ATTR amyloidosis from Europe (*n* = 16), North America (*n* = 26), Asia (*n* = 5), and Australia (*n* = 2). No studies were found that exclusively focused on ATTR amyloidosis in Africa or South America. ATTR prevalence ranged from 6.1/million in the US to 232/million in Portugal with very limited data on ATTR-PN. The 2-year mortality risk ranged from 10 to 30% among wild-type ATTR-CM and from 10 to 50% for variant type of ATTR-CM.

**Conclusions:**

This SLR demonstrated heterogeneity in ATTR epidemiology and mortality rates across global regions. Further investigation is needed to address knowledge gaps of the epidemiology and burden of ATTR, which may improve early diagnosis and management.

**Supplementary Information:**

The online version contains supplementary material available at 10.1186/s13023-025-03547-0.

## Introduction

Transthyretin (ATTR) amyloidosis is a rare, progressive disease characterized by the abnormal accumulation of amyloid deposits, composed of misfolded transthyretin protein, in the body’s organs and tissues [1, 2]. Recent data suggest that the prevalence of ATTR amyloidosis may be as high as 15% in patients with cardiovascular comorbidities [3–11]. Furthermore, the prevalence of ATTR amyloidosis notably varies by genotype and phenotype. Certain genetic mutations that are linked to transthyretin protein misfolding are more prevalent in specific populations [12]. Phenotypic expressions of the disease vary widely [13, 14], influencing both clinical presentation and disease progression.

Diagnosing ATTR amyloidosis can be challenging because of its nonspecific symptoms, which may present similarly to other medical conditions (e.g., diabetic neuropathy, chronic inflammatory demyelinating polyneuropathy [PN], heart failure (HF), and other cardiac conditions) [12]. ATTR amyloidosis is fatal if diagnosis and treatment initiation are delayed with a life expectancy between 3 and 15 years after the onset of symptoms; therefore, early identification, diagnosis, and treatment are critical [15]. Advances in diagnostic methods and the increasing availability of targeted treatments [16] have enhanced the early detection and timely management of ATTR amyloidosis in recent years and have fueled interest in the improved understanding of characteristics of patients with ATTR amyloidosis and related management strategies. Despite these advances, barriers to early diagnosis and accessibility of treatment options remain, particularly in healthcare settings with relatively fewer resources [17, 18]. Although previous systematic literature reviews (SLRs) on the epidemiology of ATTR amyloidosis have advanced our understanding, they have focused primarily on specific ATTR amyloidosis phenotypes or have been conducted within high-risk groups or manifestations of the disease [14, 19–23], leading to a fragmented view. This segmented approach leaves a significant gap in our collective knowledge of ATTR amyloidosis and underscores the need for a more expansive and integrative systematic review. Therefore, the overarching goal of this review was to systematically summarize contemporary literature on the epidemiology and mortality rates of ATTR amyloidosis and filling this critical void in the existing body of literature.

## Methods

### Search strategy and criteria

This SLR was conducted in accordance with the Preferred Reporting Items for Systematic Reviews and Meta-analyses (PRISMA) guidelines [24]. Systematic literature searches were conducted in OvidSP to identify peer-reviewed studies of interest published between January 2018 and April 2023 in MEDLINE and Embase databases. The search strategies (i.e., specific keywords and MeSH terms used) are detailed in Table S1.

### Inclusion and exclusion criteria

The inclusion and exclusion criteria were predefined in a patient, intervention, comparator, outcome, time, study (PICOTS) table during protocol development (Table S2). The scope of inclusion encompassed studies evaluating populations diagnosed with various manifestations of ATTR amyloidosis, including both variant and wild-type classifications in cardiomyopathy (CM) and PN, as well as mixed phenotypes. Publications reporting data only from patients diagnosed with amyloidosis other than ATTR amyloidosis were excluded. Included studies were observational studies, encompassing prospective and retrospective designs; longitudinal studies with national representation; database or registry studies; as well as SLRs and meta-analyses. Both full-length articles and conference abstracts were included. We placed no restrictions on the geographical scope of the published literature, but we limited our selection to articles published in the English language. Excluded from this review were reports, letters, commentaries, editorials, clinical trials, and any studies that did not conform to the predetermined inclusion criteria. Two independent reviewers (FM & DS) screened abstracts and full text for inclusion, and a third independent reviewer (FD) resolved any discrepancies.

### Data collection and data extraction

The following information were extracted and entered into data extraction form: (1) study characteristics: title, author, publication year, study design, country, and study period; (2) population characteristics: study population, sample size, demographic data such as age and sex, ATTR amyloidosis phenotype; (3) epidemiological measures: prevalence and incidence rates of ATTR amyloidosis (by phenotype and genotype); (4) and mortality rates (Table S2). The data extraction was performed by one reviewer (FM) and cross-checked by another (FD & DS) to ensure completeness and accuracy of data. Any discrepancies encountered during this process were resolved by a third reviewer.

## Results

**Fig. 1:**
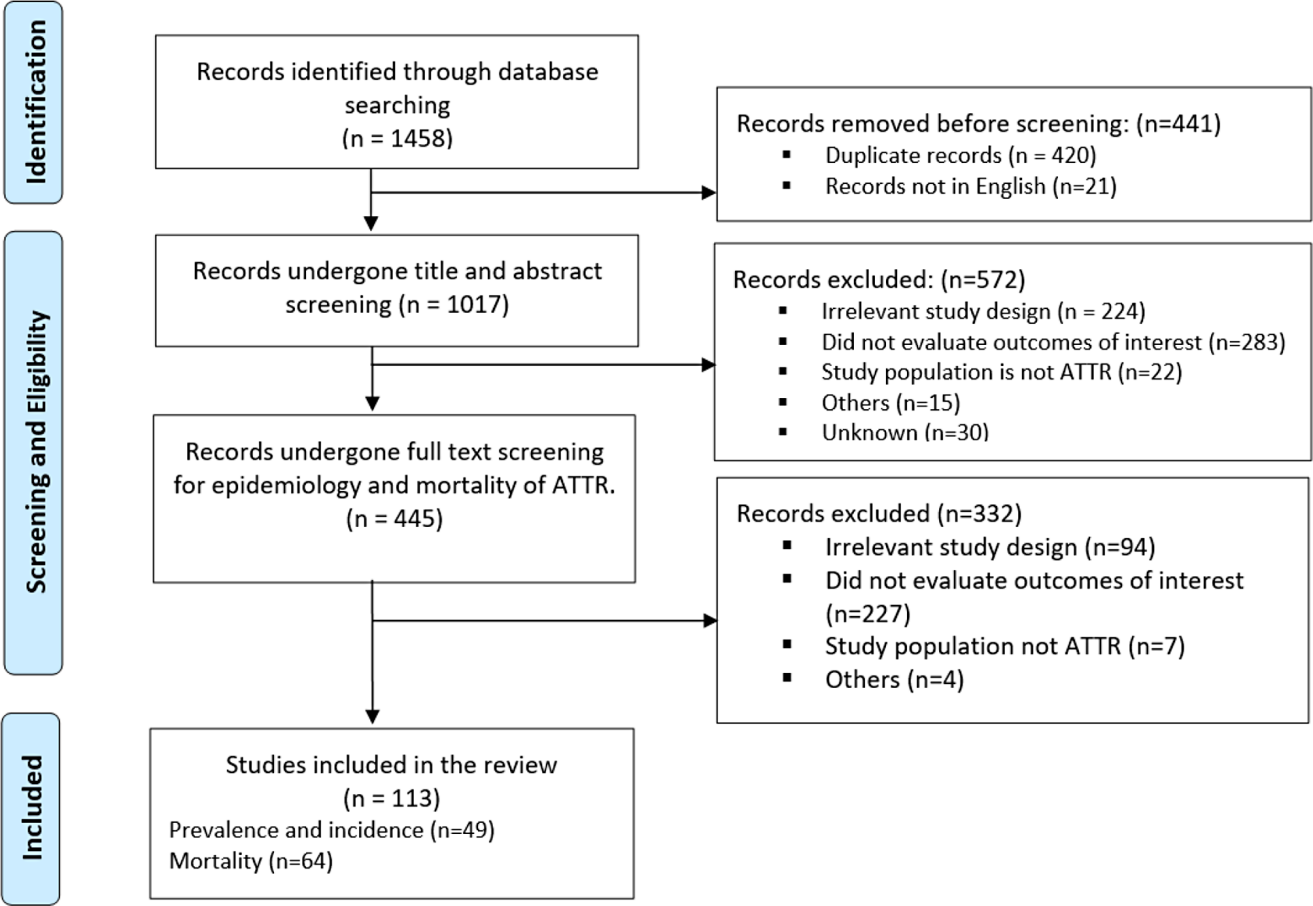
PRISMA flow chart summarizing the selection process of articles included in this systematic review. Abbreviations: ATTR = transthyretin amyloidosis; CM = cardiomyopathy; NYHA = Ner York Heart Association classification; PN = polyneuropathy; PRISMA = Preferred Reporting Items for Systematic Reviews and Meta-Analyses

The database searches identified 1,458 records, of which 1,017 underwent title and abstract screening after removing duplicates. Of the abstracts reviewed, 445 were selected for full-text screening, resulting in 113 publications reporting at least one outcome of interest (Fig. 1). Of the 113 full-text publications included, 49 reported on the epidemiology of ATTR amyloidosis and 64 reported on mortality rates among patients with ATTR amyloidosis. For prevalence and incidence, most publications were from North America (n=26) and Europe (*n* = 16) , five were from Asia, and two were from Australia. No publications were found that exclusively focused on ATTR amyloidosis within Africa or South America.

### Demographic characteristics of study populations

Across studies examining the prevalence and incidence of ATTR amyloidosis in the general population, sample sizes spanned from 174 to over 10 million participants. Male representation varied between 56% and 94%, with a notable predominance of male participants across cohorts (Table S3). For example, Inês et al. 2018 (*n* = 8,133,909) reported 56% male participants, while Lauppe et al. (2021) (*n* = 10,230,185) observed 70% in a much larger sample of Sweden. The mean age of participants ranged from 52.3 ± 15.4 years in prevalent cases (Ines et al. 2018) to 83 ± 6.49 years (Manning et al. 2020), with some studies, like Damy et al. (2021), providing more detailed age stratification by sex.

For studies focusing on the ATTR amyloidosis by genotype, sample sizes were generally smaller, ranging from 40 to 2,029 participants (Table 1). Wild type ATTR-CM (ATTRwt-CM) was predominantly studied, with male participants making up between 73% and 96.5% of the cohorts. Mean ages varied from 68.8 years to 88 years for ATTRwt-CM, while patients with variant type ATTR-CM (ATTRv-CM) were relatively younger, with a mean age of 68 years.

**Table 1:** Clinical studies reporting the proportion of patients with ATTR amyloidosis by Genotype (ATTRv-CM and ATTRwt-CM)

Study	Country	Study Population	Case Identification Method	Sample Size	ATTR type (Phenotype/ Genotype)	Proportion (%)	Men	Mean age (years)
Basdavanos et al. 2023 [77]	US	CA	biopsy or cardiac imaging + genetic testing	1310	ATTRwt-CM	36.60%	77.5%	72.3
					ATTRv-CM	14.00%		
					Untyped ATTR	12.80%		
Dobner et al. 2023 [88]	Switzerland	ATTR-CM	99mTc-DPD or biopsy	57	ATTRwt-CM	94.60%	96.5%	79.2
					ATTRv-CM	5.40%		
Harapoz et al. 2022 [89]	Australia	ATTRwt/ATTRv/asymptomatic TTR variant carriers	Echocardiographic exam/99mTc-DPD	40	ATTRwt-CM	42.50%	75%	68.8
					ATTRv-CM	57.50%		
Oghina et al. 2021 [90]	France	ATTR-CM	Scintigraphy/gammopathy/biopsies	454	ATTRv-CM	30.00%	82%	77
					ATTRwt-CM	70.00%		
Patel et al. 2022 [91]	UK	ATTR-CM	HF + biopsy	1732	ATTRwt-CM	63.40%	85.9%	78.1
					ATTRv-CM	36.60%		
Porcari et al. 2022 [92]	UK	Elderly patients (aged ≥ 70) with ATTR-CM	Clinical evaluation/biochemical tests/ Echo/TTR genotyping	2029	ATTRwt-CM	79.50%	86.8%	79 (median)
					ATTRv-CM	20.80%		
Prasad et al. 2023 [53]	USA	ATTR-CM	Biopsy/echo/immunohistochemistry/CMR	140	ATTRwt-CM	80.70%	88.6%	Male: 75.8 Female: 76.6
					ATTRv-CM	19.30%		
Silverii et al. 2022 [93]	Italy	ATTRv or ATTRwt	Cardiological evaluation and CPET	75	ATTRwt-CM	76.00%	91%	80 (median)
					ATTRv-CM	24.00%		
Trimarchi et al. 2021 [94]	Italy	ATTR-CM	-	381	ATTRwt	60.00%	80.8%	–
					ATTRv	40%		
Trujillo & Colombo 2022 [95]	USA	ATTR-CM	Biopsy/ICD-10 code/SPECT + PYP protocol	144	ATTRv-CM	36.00%	73%	68
					ATTRwt-CM	64.00%	96%	88

Abbreviations: ATTR-CM = Transthyretin Amyloid Cardiomyopathy; ATTRv = Variant Transthyretin Amyloidosis; ATTRv-CM = Variant Transthyretin Amyloid Cardiomyopathy; ATTRwt = Wild-Type Transthyretin Amyloidosis; CA = Cardiac Amyloidosis; CMR = Cardiac Magnetic Resonance; CPET = Cardiopulmonary Exercise Testing; Echo = Echocardiogram; HF = Heart Failure; ICD-10 code = International Classification of Diseases, Tenth Revision code; SPECT + PYP = Single Photon Emission Computed Tomography with Pyrophosphate; TTR = Transthyretin; ATTRwt = Wild-Type Transthyretin Amyloidosis; ATTRwt-CM = Wild-Type Transthyretin Amyloid Cardiomyopathy

In studies investigating ATTR amyloidosis prevalence in heart failure populations, sample sizes ranged widely, from smaller studies with 45 participants to large cohorts of over 205,000. Male representation fluctuated from as low as 39% to a full 100% in some studies. Mean ages were typically in the late 70s, though individual studies reported ages from 65 to 81 years.

### Prevalence and incidence of ATTR amyloidosis in the general population

**Fig. 2:**
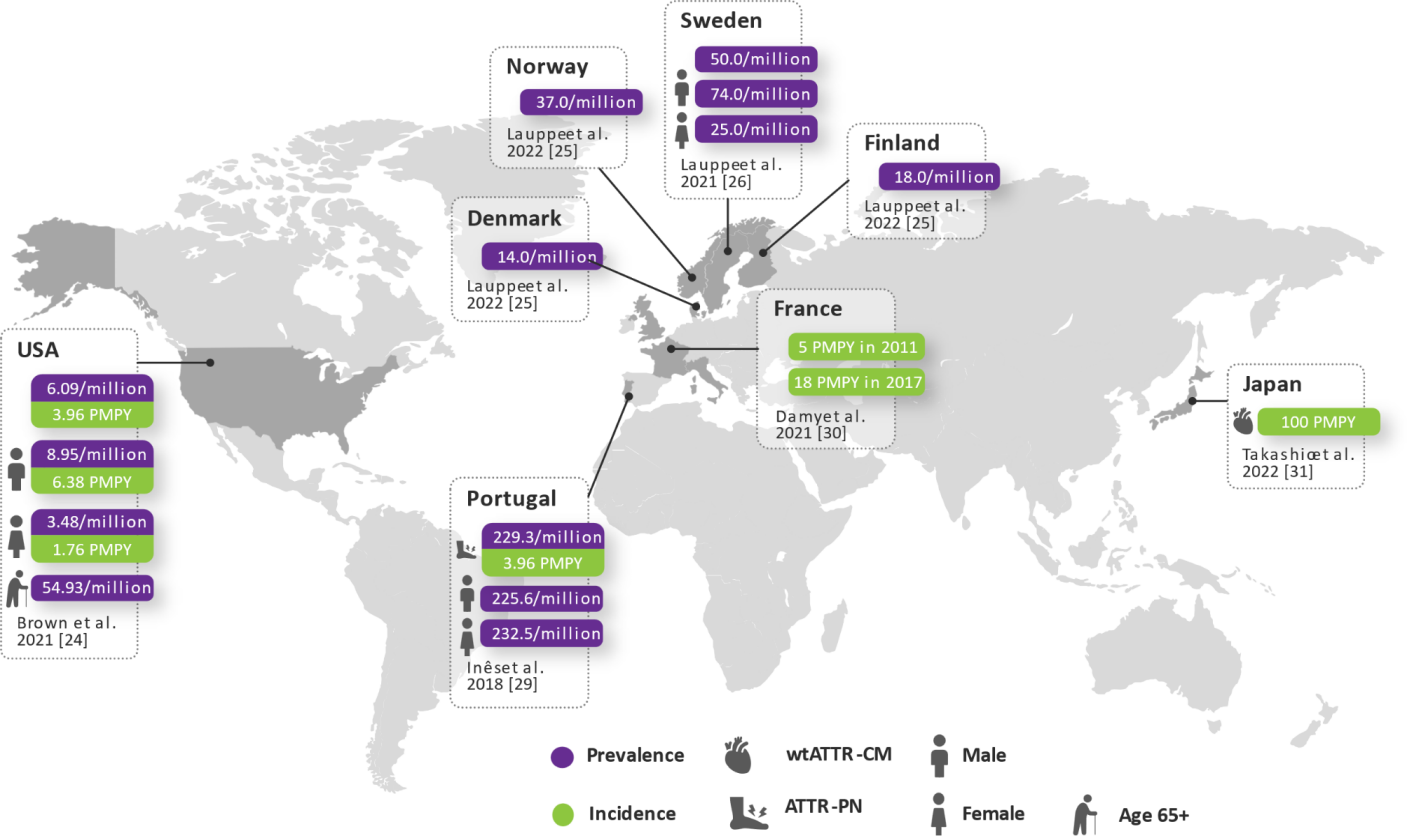
Prevalence of ATTR amyloidosis in the General Population. Except for the data from Japan and Portugal, all other prevalence and incidence rates depicted in this figure pertain to unspecified ATTR-CM. Abbreviations: ATTR-PN = amyloid transthyretin polyneuropathy; PMPY = Per Million Per Year; ATTRwt-CM = wild-type amyloid transthyretin cardiomyopathy

Six publications reported on the prevalence of ATTR-CM (without specification of variant or wild-type) in the general population (Fig. 2 and Tables S3) with significant variation across countries. In the US, the prevalence of ATTR-CM was reported to be approximately 6.1 per million [25], whereas the reported figures from Denmark (14.0 per million), Finland (18.0 per million), Norway (37.0 per million), Sweden (50.0 per million), and Japan (100.0 per million per year) were considerably higher [26, 27]. A variation in figures was also observed among the elderly (≥ 65 years old), with one study in the US using claims data reporting a prevalence of 54.9 per million [25]. Regarding ATTR-PN, the only available estimate was from a single study conducted in Portugal, where the prevalence was found to be 229.3 per million adult inhabitants [28].

Four studies reported data on the incidence of ATTR amyloidosis. One study from France, conducted using the French National Healthcare Data System [Système National Des Données De Santé (SNDS)] data set and included patients ≥ 50 years of age, reported an increase in the incidence of ATTR-CM from 5.0 to 18.0 per million person-years (PMPY) between 2011 and 2017 [29]. In the US, the incidence of ATTR-CM in 2018 was found to be 3.9 PMPY, and the highest rates were observed among elderly individuals, at 36.6 PMPY [25]. A much higher incidence rate of wild-type ATTR (ATTRwt) was reported in Japan, estimated at approximately 100 PMPY in the elderly population [30]. Additionally, an annual incidence of ATTR-PN of 8.7 PMPY was reported in Portugal [28].

### Prevalence of ATTR-CM by genotype [ATTR with variant (Hereditary) and wild-type ATTR-CM

Ten studies reported on the prevalence of ATTR-CM by genotype, including patients diagnosed with ATTR-CM who underwent additional diagnostic evaluations to differentiate ATTR with ATTRv and ATTRwt-CM forms. The prevalences of ATTRv-CM and for ATTRwt-CM varied widely across different studies, ranging from 5.4 to 57.5% and 36.6–94.6%, respectively, as shown in Table 1.

### Prevalence of ATTR-CM in patients with high-risk conditions

#### Heart failure

**Fig. 3:**
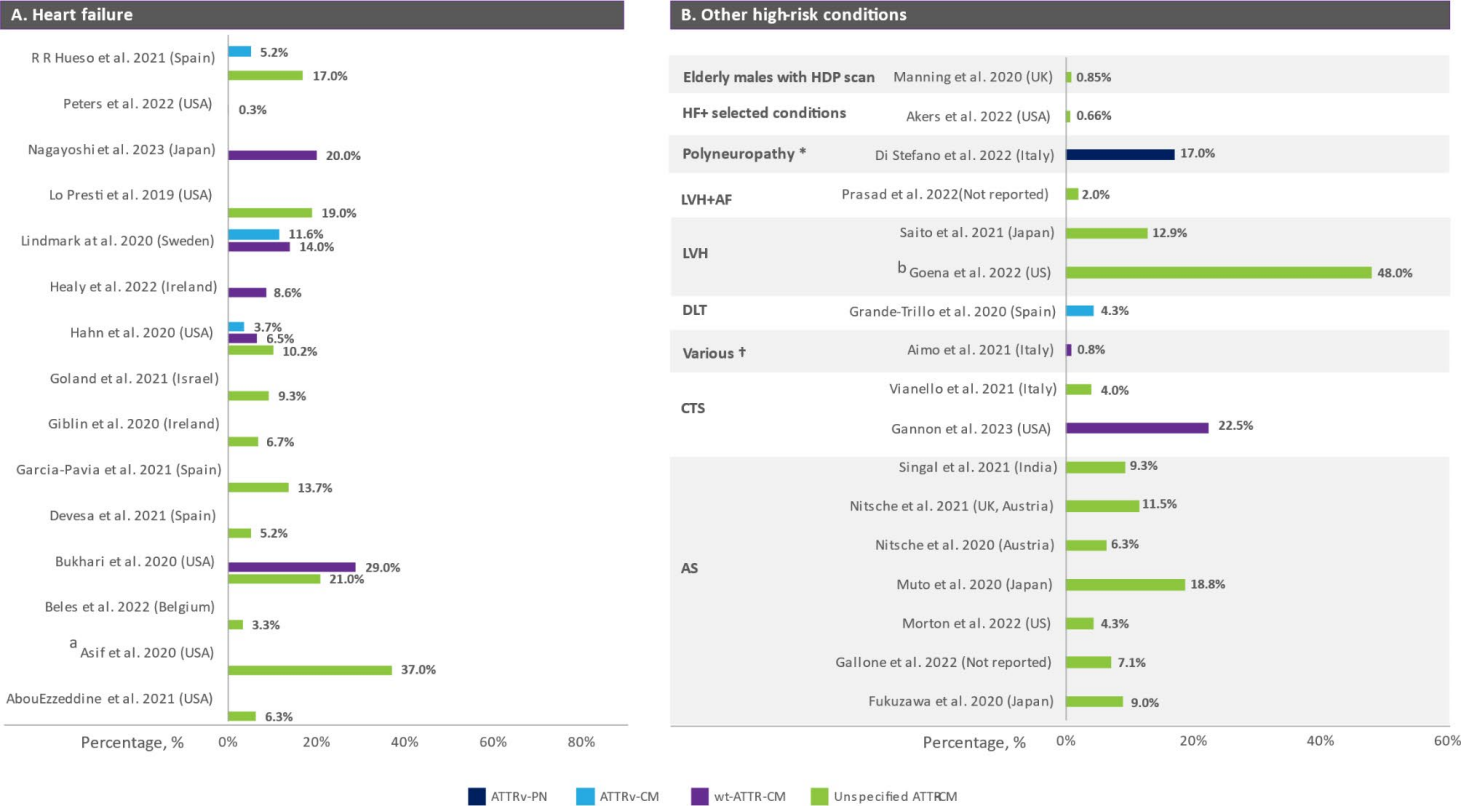
Clinical Studies Reporting the Number Per Million of ATTR amyloidosis in Patients with (**A**) Heart Failure and (**B**) other high-risk conditions. ^a^ Observed prevalence may be related to the small sample size (*n* = 87) and the specialized diagnostic criteria used. ^b^ observed prevalence was among patients suspected of ATTR amyloidosis, selected based on specific cardiac amyloidosis indicators and imaging results. *History of (CTS, LSS, etc.)/echocardiographic red flags/hs-troponin T higher than the upper reference limit. †Sensory-motor idiopathic PN and 2 + red-flag (e.g., Family history of PN or CM, CTS, etc.). Abbreviations: AF = Atrial Fibrillation; AS = Aortic Stenosis; ATTR-CM = Transthyretin Amyloid Cardiomyopathy; ATTRv-CM = Variant Transthyretin Amyloid Cardiomyopathy; ATTRv-PN = Variant Transthyretin Polyneuropathy; CTS = Carpal Tunnel Syndrome; DLT = Domino Liver Transplant; HDP = hydroxymethylene diphosphonate; HF = heart failure; LVH = Left Ventricular Hypertrophy; LVH + AF = Left Ventricular Hypertrophy plus Atrial Fibrillation; ATTRwt-CM = Wild-Type Transthyretin Amyloid Cardiomyopathy

Fifteen publications [11, 31–44] provided insights into the prevalence of ATTR-CM among patients diagnosed with HF without other underlying high-risk conditions associated with ATTR amyloidosis. These publications distinguished between unspecified ATTR-CM, ATTRv-CM, and ATTRwt-CM, revealing a nuanced landscape of ATTR amyloidosis prevalence within this high-risk group (Fig. 3a and Table S4). Of these publications, six reported specifically on the prevalence of ATTRwt-CM in patients with HF. The prevalences varied across different cohorts with various sample sizes. One study, conducted in a large sample (*n* = 205,545) of patients who were admitted for HF, reported a prevalence of 0.31% [42]. Conversely, other studies with smaller sample sizes reported higher prevalence proportions [38, 39, 41, 44] (Table S4). Regarding ATTRv-CM, insights were provided in two studies indicating that 3.7- 5.2% of patients with ATTR-CM had a hereditary cause [37, 43].

The prevalence of unspecified ATTR-CM in patients with HF was reported in 11 studies, and for most of the studies it ranged from 3.3 to 21%. However, one study that was conducted in a cohort of 87 patients with HF and included patients with both preserved and reduced ejection fraction referred for Technetium-99 m pyrophosphate (TC-PYP) imaging, reported a prevalence of 37% [31].

### Prevalence of ATTR-CM in patients with other high-risk conditions

Seventeen publications reported on the prevalence of ATTR-CM in various patient populations known to be at higher risk for ATTR amyloidosis, two were in carpal tunnel syndrome (CTS) [45, 46], seven in aortic stenosis (AS) [5, 6, 47–51], four in left ventricular hypertrophy (LVH) [52–54], and one in each of the following: sensory-motor idiopathic PN [55], domino (sequential) liver-transplant recipients [56], elderly patients with selected conditions commonly associated with ATTR-CM [57] and elderly males undergoing routine bone scans [58] (Fig. 3b and Table S5). The prevalence of ATTR-CM in most of the studies was highly variable in patients with the aforementioned risk factors (Fig. 3b and Table S5).

### Mortality outcome of transthyretin amyloidosis

Our review identified 64 studies reporting all-cause mortality in patients with ATTR amyloidosis; aggregated data are presented in Table S6. These studies exhibit variability in their reporting approaches, some focusing on mortality rates at specific time points (e.g., 1 year, 2 years, or 5 years), others on median survival times, and yet others on overall survival rates. Median survival times diverged among these studies, ranging from 12 to 80 months. In 50 of 52 studies that reported data on age and sex, the patient population was predominantly elderly men. There was apparent variability in mortality rates across different population types and by geography. The 2-year mortality risk for ATTRwt-CM and ATTRv-CM ranged from 10 to 30% and 10–52%, respectively (Table 2).

**Table 2:** Clinical studies on all-cause mortality rates of among patients with ATTR

Author	Year	Country	Subgroup	Size (*n*)	Median Follow–up (months)	Median Survival (months)	Mortality risk				Age (years)	Male Sex
							1 year	2 years	5 years	Overall		
Ali et al. 2020 [96]	2020	USA	ATTR	357	20.4	–	–	47%	–	–	76	–
Amadio et al. 2022 [97]	2022	USA	ATTR - CM	2539	–	–	–	–	–	–	67	71%
Amaka et al. 2022 [81]	2022	USA	ATTRwt overall	108	60	–	–	–	49%	–	–	–
Arana et al. 2022 [98]	2022	Spain	ATTR	266	13	–	50%	–	–	–	77.7	90%
Bandera et al. 2022 [99]	2022	UK	ATTR - no SR	342	–	51	2%	28%	70	–	76.9	92%
Bhattacharya et al. 2022 [100]	2022	USA	ATTR	200	180	–	–	–	–	65%	–	–
Bukhari et al. 2020 [101]	2020	USA	ATTR	124	18	–	–	–	–	22%	87	80%
Bustillo et al. 2020 [102]	2020	Spain	ATTR	44	14	–	–	–	–	14%	83	77%
Chacko et al. 2020 [103]	2020	UK	ATTR - CM	1240	–	–	–	23	–	39%	–	–
Dalia et al. 2022 [104]	2022	USA	ATTRwt - exercise duration > 5.5 min	11	12	–	12%	–	–	–	82	82%
			ATTRwt - Exercise duration ≤ 5.5 min	22	12	–	33%	–	–	–	82	77%
Damy et al. 2021 [29]	2021	France	ATTR	4815	84	33.7	31%	50%	–	–	80	67%
Donnellan et al. 2020a [105]	2020	USA	ATTR - CM	–	–	–	–	–	–	60%	–	–
Donnellan et al. 2020b [106]	2019	USA	ATTR - AF ablation	24	–	–	4%	22%	–	–	74	96%
			ATTR - no AF ablation	48	–	–	25%	32%	–	–	–	–
Driggin et al. 2020 [107]	2020	USA	ATTR - mBMI high	73	–	–	3%	11%	32%	–	–	–
			ATTR - mBMI low	182	–	–	10%	24%	58	–	76	86%
Gonzalez-Lopez et al. 2022 [108]	2022	Spain, Italy, France, Finland, and US	ATTR - CM	118	44.4	–	5	–	18%	–	66	78%
Hein et al. 2021 [109]	2021	Germany	ATTR - CM	89	14.8	–	–	–	–	10%	–	–
Hoerbrand et al. 2023 [110]	2023	Germany	ATTR patients with Tricuspid regurgitation undergoing TTVR	–	3	–	–	–	–	0%	80	75%
Hussain et al. 2022a [111]	2022	USA	ATTR-CM	580	~ 36	–	–	–	–	53%	74	69%
Jang et al. 2022 [112]	2022	South Korea	ATTR-CM	715	132	18	–	–	–	35%	69.3	62%
Lauppe et al. 2022 [26]	2022	Sweden	ATTR-CM	1930	132	30	–	–	–	–	73	69%
Lauppe et al. 2021 [27]	2021	Sweden	ATTR	994	–	–	24%	38%	64	–	73	70%
Leon Cejas et al. 2020 [113]	2020	Argentina	ATTR-PN	94	–	–	–	–	–	13%	35	52%
Nativi-Nicolau et al. 2020 [114]	2020	USA	ATTR-CM	894	6	–	–	–	–	22	–	63%
Nitsche et al. 2020 5]	2020	Austria	ATTR	15	–	–	20%	–	–	–	84	63%
Oghina et al. 2021 [90]	2021	France	ATTR – NT-proBNP increasing	121	–	–	58%	80%	–	–	–	–
			ATTR – NT-proBNP stable	333	14.2	–	41%	62%	–	–	77	82%
Rashdan et al. 2020 [115]	2020	USA	ATTR-CM	91	~ 28	–	–	–	–	28%	73	92%
Righetto et al. 2021 [116]	2021	Italy	ATTR	67	–	–	–	–	–	60	–	75%
Salvalaggio et al. 2022 [117]	2022	Italy	ATTR	67	47	–	–	–	–	61%	78	65%
Shimoni et al. 2021 [118]	2021	Israel	ATTR	11	38	–	13%	13%	–	–	81.7	7%
Silverii et al. 2022 [93]	2022	Italy	ATTR-CM	75	~ 25	–	15%	–	–	16%	80	91%
Slama et al. 2020 [119]	2020	France	ATTR-CM	4815	24	33.7	28%	–	–	36%	78	67%
Sperry et al. 2018 [120]	2018	USA	ATTR-CM	54	21.48	–	–	–	–	48%	78	76%
Uusitalo et al. 2022 [121]	2022	Finland	ATTR	17	12	–	–	–	–	88%	80	78%
Vranian et al. 2018 [122]	2018	USA	ATTR	48	–	–	–	–	–	56%	78	73%
Zhang et al. 2020 [123]	2020	USA	ATTR	136	24	–	–	–	–	49%	–	–

Abbreviations: AF = Atrial Fibrillation; ATTR = Transthyretin Amyloidosis; ATTR-CM = Transthyretin Amyloid Cardiomyopathy; mBMI = Modified Body Mass Index; NT-proBNP = N-terminal pro b-type Natriuretic Peptide; SR = Sinus Rhythm; TTVR = Transcatheter Tricuspid Valve Repair; ATTRwt = Wild-Type Transthyretin Amyloidosis

## Discussion

This SLR provides contemporary insights into the global understanding of the epidemiology of ATTR amyloidosis in the general population and in specific high-risk populations. Our review highlights the limitation in research that offers comprehensive global ATTR-specific epidemiology and mortality data thus hindering our understanding of its true global burden. Given the observed heterogeneity in study designs, an evaluation of each setting presented challenges as many of the studies included in this review were conducted within specific clinical cohorts and with variation in sample sizes, which may have introduced potential selection bias. These variations could influence the reported prevalence and incidence rates, potentially skewing the true impact of ATTR amyloidosis in the general population.

Despite the significant advances in non-invasive diagnostic techniques for ATTR amyloidosis [59] such as enhanced imaging modalities [60, 61], timely diagnosis remains a formidable barrier to prompt treatment initiation. Some cardiologists persist in their belief that ATTR amyloidosis is untreatable and might be hesitant to pursue additional invasive testing for accurate diagnosis. In addition, the inadequate dissemination of diagnostic advancements and updated techniques to healthcare professionals often result in patients encountering delayed diagnosis of several years [62–64]. This delay could also be attributed to the subtle onset and the nonspecific nature of early symptoms [64]. Overall, the prevalence of ATTR-CM can be described as rare in accordance with regulatory definitions for orphan disease [65, 66]. Our findings are consistent with previous meta-analyses, which also reported an increased prevalence of ATTR-CM in populations with HF and AS, albeit with notable heterogeneity across all subgroups [21].

The high prevalence of ATTR amyloidosis in patients with HF and AS suggests that routine screening in these groups may yield a significant number of undiagnosed cases, thereby facilitating earlier intervention and potentially altering the disease course [67, 68]. Our findings also highlight the critical role of clinical vigilance and awareness of risk factors symptoms in identifying ATTR amyloidosis [69–72]. These risk factors, including LVH, CTS, and neuropathy, are important clues to prompt clinicians to consider ATTR amyloidosis in their differential diagnosis. Identifying cost-effective strategies for ATTR-CM diagnosis in different clinical settings remains a priority [73]. Large-scale prospective studies, such as the AC-TIVE study [74], which highlighted the importance of systematic screening for cardiac amyloidosis risk factors in routine echocardiography in patients who were ≥ 55 years of age, are important to understand burden of disease.

Many studies included in this review originated in Europe and North America, and there was a notable absence of data from regions such as Africa and South America, suggesting geographical disparity in ATTR research. It is worth mentioning that regions such as South America, particularly Argentina and Brazil, have been previously identified as endemic areas for ATTR amyloidosis. Data from global studies like the Transthyretin Amyloidosis Outcomes Survey (THAOS) confirms the presence of the disease in these regions [75], indicating that ATTR amyloidosis is indeed a global concern. Another significant concern is the absence of clinical registries and cohorts for ATTR amyloidosis in much of the world, notably outside Europe and the US. This gap underscores the necessity for establishing clinical registries in regions such as Africa, Asia, and South America to better understand the true burden of the disease in these regions. In our review, only two studies reported differential estimates of ATTR amyloidosis by race or ethnicity [44, 76]. These limited data, which suggest worse outcomes in ethnic minorities [77], point to a significant research gap regarding the impact of ATTR amyloidosis among these cohorts. Further research to elucidate the racial disparity is essential for ensuring equitable access to effective treatments and improving health outcomes across all populations affected by this condition [76].

The variability in diagnostic criteria used in clinical practice emerged as a significant observation in our review. This absence of consistency complicates comparison of findings across studies and may contribute to the underdiagnosis or misdiagnosis of ATTR amyloidosis [78]. TC-PYP scan, or scintigraphy using hydroxy diphosphonate or diphosphonate (DPD), are affordable and highly accurate methods to confirm the diagnosis of ATTR-CM. However, confirmatory testing with 99mTc-PYP imaging [79] and recommended monoclonal protein evaluation before PYP scanning [80] remain significantly underused. Standardized diagnostic criteria along with awareness of the disease are essential for improving the accuracy of ATTR amyloidosis diagnosis and for facilitating research that can be more easily compared and synthesized.

The mortality trends observed in our review of patients with ATTR amyloidosis underscore the complex interplay of factors influencing patient outcomes. Notably, the variability in survival rates at different time intervals highlights a critical aspect of ATTR amyloidosis progression. A notable limitation of our mortality data lies in the inconsistency of reporting across the studies, compounded by variable duration of follow-up and differences in population characteristics. The initial higher survival rates, which decline over longer periods, particularly in the 5-year data for patients with ATTRwt, point to the progressive nature of the disease and the potential for late-stage complications [81]. This trend underscores the importance of early diagnosis and intervention, which could potentially alter the disease trajectory. The marked improvement in survival rates with emerging therapies [82, 83] suggest that advancements in treatment options have the potential to significantly improve life expectancy and quality-of-life for ATTR amyloidosis patients.

Our review highlights a notable scarcity of studies focused on ATTR-PN, particularly those addressing patients with ATTR amyloidosis with mixed phenotype, in which both PN and CM manifestations are present. The analysis of the THAOS registry recently reported that approximately one-third of symptomatic ATTR amyloidosis patients (*n* = 1,185/3,542; 33.5%) were classified as mixed phenotype, suggesting this may be more prevalent than previously thought [84]. The true global prevalence of ATTR-PN remains unclear, though it is estimated to be between 10,000 and 40,000 persons globally, with endemic foci in Portugal, Sweden, and Japan [85] with the vast majority (84%) diagnosed with Coutinho disease Stage 1 (ambulatory) [86]. These findings highlight a significant gap in our understanding of ATTR amyloidosis as a multisystem disease. In addition, the inconsistency in the application of genetic testing in confirmed cases of ATTR-CM is another constraint representing missed opportunities for familial risk assessment and intervention [87].

Although this review provides a broad perspective on ATTR amyloidosis, it is not without limitations. The reliance on published literature may introduce publication bias, and the lack of representation from certain regions limits the generalizability of the findings. In addition, the heterogeneity of the data prevented the execution of meta-analyses due to a low number of studies sharing similar characteristics such as the phenotype of ATTR amyloidosis or the presence of any high risk condition. A careful examination of any possibility to perform a meta-analysis resulted consistently in a too large difference between the investigated sub-populations to make this feasible. As a result, the absence of meta-analyses hampers the interpretation of the overall findings. However, despite these limitations, the strength of this review lies in its extensive coverage, contemporariness, and inclusion of a diverse range of study designs and patient populations.

Conclusion: In conclusion, our findings demonstrate that ATTR amyloidosis remains a rare disease, displays heterogeneity in its epidemiology across geographic regions and populations studied. Higher prevalence/incidence of ATTR amyloidosis was observed among men, the elderly, and those with cardiac conditions, thereby suggesting potential target populations to screen for timely diagnosis and treatment initiation. Robust epidemiological ATTR amyloidosis data requires further investigation in a larger sample of patients with diverse phenotypes across multiple countries and world regions.

## Electronic supplementary material

Below is the link to the electronic supplementary material.

Supplementary Material 1

## Data Availability

Not applicable.
