# Supplementary material for: Epidemiology of transthyretin (ATTR) amyloidosis: a systematic literature review
Source: Orphanet J Rare Dis. 2025 Jan 16;20:29. doi: 10.1186/s13023-025-03547-0 (PMC11740649; doi:10.1186/s13023-025-03547-0)
Supplement: Supplementary file 1 — Supplementary Material 1 [file 13023_2025_3547_MOESM1_ESM.docx]

**Supplementary material**

Epidemiology of Transthyretin (ATTR) Amyloidosis: A Systematic Literature Review

Contents

[**Table S1**: Ovid® Search Strategies for EMBASE and Medline and Number of Citations Identified 2](#_Toc164326739)

[**Table S2**. PICOTS Criteria for Study Inclusion and Exclusion in the SLR 4](#_Toc164326740)

[**Table S3**: Clinical Studies Reporting the Prevalence of ATTR in the General Population. 5](#_Toc164326741)

[**Table S4**: Clinical Studies Reporting the Prevalence of ATTR in Patients with Heart Failure 7](#_Toc164326742)

[**Table S5**: Clinical Studies Reporting the Prevalence of ATTR in Patients with various High-risk Conditions. 9](#_Toc164326743)

[**Table S6:** Clinical studies on all-cause mortality rates of among patients with ATTR: detailed exposition across all study groups and/or conditions 11](#_Toc164326744)

[**References:** 16](#_Toc164326745)

# **Table S1**: Ovid® Search Strategies for EMBASE and Medline and Number of Citations Identified

| **Search Number** | **Search Terms** | **Results** |
| --- | --- | --- |
| **1** | (transthyretin amyloid cardiomyopath$ or transthyretin amyloid or ((cardiomyopath$ or cardiac) adj3 (transthyretin amyloidosis or ATTR-amyloidosis or TTR-related amyloidosis or ATTR or ATTR-CM))).ti,ab. | 2504 |
| **2** | (hereditary ATTR amyloidosis with polyneuropathy or hereditary transthyretin amyloidosis with polyneuropathy or transthyretin familial amyloid polyneuropathy or hATTR-PN or TTR-FAP or familial amyloid polyneuropathy or Wildtype ATTR amyloidosis with polyneuropathy or Wildtype transthyretin amyloidosis with polyneuropathy or wATTR-PN or ATTR-PN).ti,ab. | 2687 |
| **3** | 1 or 2 | 5069 |
| **4** | 3 not (animals/ not humans/) | 5055 |
| **5** | 4 not ((exp animal/ or nonhuman/) not exp human/) | 5013 |
| **6** | case reports/ or case study/ or case report$.jx. or case report$.jw. or (case report or case study or woman or man or child or adolescent or female or male or boy or girl or infant or unusual case).ti. | 4140219 |
| **7** | (Ephemera or "Introductory Journal Article" or News or "Newspaper Article" or Editorial or Comment or Overall or Letter or Short Survey or Tombstone or Books).pt. or in vitro Techniques/ or in vitro study/ or (commentary or editorial or comment or letter or mice or rat or mouse or animal or murine).ti. | 9243227 |
| **8** | 5 not (6 or 7) | 4312 |
| **9** | exp retrospective study/ or exp observational study/ or exp Retrospective Studies/ or exp electronic health record/ or exp electronic health records/ or exp electronic medical record/ or exp cross-sectional study/ or exp Cross-Sectional Studies/ or (retrospective study or observational study or real-world or cross-sectional or electronic health record$ or EHR or electronic medical record$ or EMR$ or RWE or integrated data or health system data or hospital data).ti,ab. | 4625181 |
| **10** | exp hospitalization/ or exp length of stay/ or (healthcare resource$ or medical resource$ or health resource consumption or health care consumption or "healthcare resource use" or hospital$ or admit or readmit or re-admit or admission or readmission or re-admission or length of stay or visit or visits or ER or emergency room or emergency department or impact or impacts or cost or procedure$ or stent or stenting or stents or defibrillator$ or dialysis$).ti,ab. | 12000661 |
| **11** | exp "costs and cost analysis"/ or exp Health Care Costs/ or exp Drug Costs/ or exp "Cost of Illness"/ or exp Hospital Costs/ or exp Economics, Pharmaceutical/ or exp health care cost/ or exp drug cost/ or exp hospital cost/ or exp Pharmacoeconomics/ or exp Disability-Adjusted Life Years/ or (cost or costs or economic or economics or Disability-Adjusted Life Years or dalys).ti,ab. | 2455659 |
| **12** | exp epidemiology/ or exp incidence/ or exp prevalence/ or (Epidemiolog$ or incidence or prevalence).ti,ab. | 7572199 |
| **13** | exp Genotype/ or exp Phenotype/ or (Genotype$ or phenotype$).ti,ab. | 2663448 |
| **14** | exp Drug therapy/ or exp patisiran/ or exp inotersen/ or exp vutrisiran/ or exp tafamidis/ or (treat$ or medication or Onpattro or patisiran or ALN-TTR02 or Tegsedi or inotersen or Vutrisiran or Tafamidis or Vyndamax or Vyndaqel or FX1006A or FX-1006A).ti,ab. or Dt.fs. | 20357840 |
| **15** | exp "quality of life"/ or exp caregiver burden/ or exp disease burden/ or (burden$ or quality of life).ti,ab. | 1918484 |
| **16** | exp mortality/ or exp morbidity/ or (mortality or morbidity or survival or death).ti,ab. | 7539424 |
| **17** | exp organ transplantation/ or (organ adj3 (failure or transplant$)).ti,ab. | 783043 |
| **18** | Exp Rankin scale/ or exp Barthel index/ or exp functional status/ or exp disability/ or (indirect cost$ or disability or functional status or physical function or impairment or disabilities or productivity or employment or retirement or work disability or absenteeism or presenteeism or sick leave or sick day or worktime loss or opportunity loss or job performance or (work adj2 loss)).ti,ab. or (Modified Rankin Scale or mRS or Barthel Index or BI or mBI).ti,ab. | 2164614 |
| **19** | (time adj2 diagnos$).ti,ab. | 122726 |
| **20** | Exp Kansas City Cardiomyopathy Questionnaire/ or (Kansas adj2 cardiomyopathy adj2 questionnaire).ti,ab. or  KCCQ-OS.ti,ab. | 2533 |
| **21** | Exp Norfolk Quality of Life Diabetic Neuropathy Questionnaire/ or ((Quality-of-Life adj2 Diabetic Neuropathy) or (Norfolk adj2 QOL adj2 neuropathy)).ti,ab. or “Norfolk QOL-DN”.ti,ab. | 291 |
| **22** | Exp European Quality of Life 5 Dimensions Visual Analogue Scale/ or exp European Quality of Life 5 Dimensions 5 Level questionnaire/ or (European adj2 Quality-of-Life adj2 Dimensions).ti,ab. or EQ-5D.ti,ab. | 36001 |
| **23** | (SF6 or SF-6).ti,ab. or (short form-6 adj2 survey).ti,ab. | 4925 |
| **24** | Exp Short Form 12/ or (SF12 or SF-12).ti,ab. or (short form-12 adj2 survey).ti,ab. | 20364 |
| **25** | Exp Short Form 36/ or (SF36 or SF-36).ti,ab. or (short form-36 adj2 survey).ti,ab. | 92965 |
| **26** | Exp Functional Independence Measure/ or (Polyneuropathy Disability score or PND or Neuropathy Impairment Scale$ or NIS-LL or Functional Independence Measure$ or FIM or ACTIVLIM$ or Compound Autonomic Dysfunction or CADT).ti,ab. | 31352 |
| **27** | or/9-26 | 36679663 |
| **28** | *adaptive clinical trial/ or *controlled clinical trial/ or *phase 1 clinical trial/ or *phase 2 clinical trial/ or *phase 3 clinical trial/ or *randomized controlled trial/ or *Clinical Trials, Phase I/ or *Clinical Trials, Phase II/ or *Clinical Trials, Phase III/ | 33414 |
| **29** | (8 and 27) not 28 | 3419 |
| **30** | limit 29 to (article or article in press) | 1930 |
| **31** | limit 29 to conference abstract | 2125 |
| **32** | limit 30 to yr=”2018-Current” | 1012 |
| **33** | limit 31 to yr=”2020-Current” | 829 |
| **34** | 32 or 33 | 1458 |
| **35** | remove duplicates from 34 | 1038 |
| **36** | limit 35 to english language | 1017 |

# **Table S2**. PICOTS Criteria for Study Inclusion and Exclusion in the SLR

| **Criterion** | **Explanation** |
| --- | --- |
| Inclusion criteria |  |
| Population | Subjects with ATTRv-CM, wtATTR-CM, ATTRv-PN, wtATTR-PN, ATTRv-mixed type, or wtATTR-mixed type |
| Intervention | N/A |
| Comparator | N/A |
| Outcomes | (1) prevalence and incidence; (2) mortality |
| Study design | Observational studies, including prospective or retrospective, nationally representative longitudinal studies or database/registry studies, systematic literature reviews, and meta-analyses. |
| Others | Geographical scope: No restriction on geographical regions  Language: Written in English |
| Exclusion criteria | |
| Study design | Reports, case reports, case series, letters, comments, and editorials |
| Others | All citations that did not meet inclusion criteria above |

Abbreviations: CM = cardiomyopathy; ATTRv = hereditary transthyretin amyloidosis; N/A = not applicable; PICOTS = patient, intervention, comparator, outcome, time, study; PN = polyneuropathy; wtATTR = wild type transthyretin amyloidosis

# **Table S3**: Clinical Studies Reporting the Prevalence of ATTR in the General Population.

| **Study** | **Country** | **Study Population** | **Case Identification Method** | **Sample Size** | **Disease Type** | **Prevalence/million** | **Incidence** | **Male (%)** | **Mean age (years)** |
| --- | --- | --- | --- | --- | --- | --- | --- | --- | --- |
| Brown et al. 2021 [25] | USA | General population using claims database | ICD-10-CM in 2018/ Another amyloidosis in 2018 +(CHF, CM, neuropathy) prior to 2018 | – | ATTR-CM | 6.09 | 3.96 PMPY | – | – |
|  |  |  |  |  | ATTR-CM in males | 8.95 | 6.38 PMPY | – | – |
|  |  |  |  |  | ATTR-CM in females | 3.48 | 1.76 PMPY | – | – |
|  |  |  |  |  | ATTR-CM in 65+ | 54.93 | – | – | – |
| Damy et al. 2021 [29] | France | SNDS (≥ 50 years of age) | ATTR-CM diagnosis required both an amyloidosis and a CV condition | – | ATTR-CM | – | 5 PMPY in 2011 and increased to 18 PMPY in 2017 | 67% | Men: 82.0 Women: 84.0 |
| Inês et al. 2018 [28] | Portugal | adult population (aged ≥18) residing in mainland Portugal | ATTR-PN patients were identified using data from two national reference centers for follow-up and clinical management | 8,133,909 | ATTR-PN | 22.93 | 8.7 PMPY | Incidence cases: 56% Prevalent cases: 46% | Incident cases: 42.8  Prevalent cases: 52.34 |
|  |  |  |  |  | ATTR-PN in males | 22.56 | – | – | – |
|  |  |  |  |  | ATTR-PN in females | 23.25 | – | – | – |
| Lauppe et al. 2021 [27] | Sweden | National population in 2018 | ICD-10-CM | Swedish population in 2018  [10,230,185] | ATTR-CM | 50.0 | – | 70% | 73 and 73.1 for women |
|  |  |  |  | Male population in 2018  [5,142,438] | ATTR-CM in males | 74.0 | – | – | – |
|  |  |  |  | Female population in 2018  [5,087,747] | ATTR-CM in females | 25.0 | – | – | – |
| Lauppe et al. 2022 [26] | Denmark | Danish National Patient Register | ICD-10-CM | Denmark population in 2018  [5,806,081] | ATTR-CM | 14.0​​ | – | – | – |
|  | Finland | Finland National Patient Register | ICD-10-CM | Finland population in 2018  [5,517,919] | ATTR-CM | 18.0​​ | – | – | – |
|  | Norway | Norwegian National Patient Register | ICD-10-CM | Norway population in 2018  [5,328,212] | ATTR-CM | 37.0​ | – | – | – |
| Takashio et al. 2022 [30] | Japan | Elderly population (aged ≥65) residing in Kumamoto Prefecture | Genetic testing | 174 | wtATTR-CM | – | 100 PMPY | 81% | 80 |

Abbreviations: ATTR-CM = amyloid transthyretin cardiomyopathy; ATTR-PN = amyloid transthyretin polyneuropathy; CATCH = Characterizing the burden of Amyloid Transthyretin CardiomyopatHy in the elderly; CHF = congestive heart failure; CM = cardiomyopathy; CV = cardiovascular; FAP = familial amyloid polyneuropathy; HDP = hydroxymethylene diphosphonate; HF = heart failure; ICD-10-CM = International Classification of Diseases, Tenth Revision; Clinical Modification; PMPY = per million per year; PY = person-years; SNDS = French National Health Data System; TTR = transthyretin; wtATTR-CM = wild-type amyloid transthyretin cardiomyopathy

# **Table S4**: Clinical Studies Reporting the Prevalence of ATTR in Patients with Heart Failure

| **Study** | **Study Population** | **Case Identification Method** | **Sample size** | **ATTR type (phenotype/genotype)** | **Proportion, (%)** | **Male (%)** | **Mean Age (years)** |
| --- | --- | --- | --- | --- | --- | --- | --- |
| AbouEzzeddine et al. 2021 [11] | HF in ≥60 years of age | HF+PYP scan | 286 | unspecified ATTR-CM | 6.30% | 51% | 80 |
|  |  |  |  | unspecified ATTR-CM in males | 10.10% |  |  |
|  |  |  |  | unspecified ATTR-CM in females | 2.20% |  |  |
| Asif et al. 2020 [33] | HFrEF and HFpEF referred Tc-PYP imaging | Perugini score ≥2 /PYP SPECT/Echo | 87 | unspecified ATTR-CM | 37% | 71.80% | 80.9 |
| Beles et al. 2022 [34] | HF patients referred to a community HF clinic | EHR using NLP | 3035 | unspecified ATTR-CM | 3.30% | 50% | 74 |
| Bukhari et al. 2020 [46] | PYP scan after HF-related hospitalization | 99mTc-PYP scintigraphy + negative serum studies | 155 | wtATTR-CM | 29% | - | 78.4 |
|  |  |  |  | unspecified ATTR-CM | 21% |  |  |
| Devesa et al. 2021 [35] | Hospital admission for HF + LVEF | 99mTc-DPD scintigraphy | 58 | unspecified ATTR-CM | 5.30% | 54% | 79 |
| Garcia-Pavia et al. 2021 [36] | HFpEF in ≥50 years of age | Clinical screening with scintigraphy | 183 | unspecified ATTR-CM | 13.70% | 52.4 | - |
| Giblin et al. 2020 [37] | HFpEF with NYHA II-IV in ≥60 years of age | 99mTc-DPD scintigraphy + negative haematological markers | 45 | unspecified ATTR-CM | 6.70% | - | - |
| Goland et al. 2021 [38] | HF with systolic dysfunction and extensive work up for ATTR | 99mTc-PYP scintigraphy | 75 | unspecified ATTR-CM | 9.30% | 100% | 65 |
| Hahn et al. 2020 [39] | HFpEF | Biopsy | 108 | unspecified ATTR-CM | 10.20% | 39% | 66 |
|  |  |  |  | wtATTR-CM | 6.50% |  |  |
|  |  |  |  | ATTRv-CM | 3.70% |  |  |
| Healy et al. 2022 [40] | HFpEF with NYHA class II-IV in >60 years of age | 99mTc-DPD scintigraphy | 81 | wtATTR-CM | 8.60% | 43% | 78 |
| Lindmark at al. 2020 [41] | HF and LVH | 99mTc-DPD scintigraphy | 86 | wtATTR-CM | 29% | 57% | 76.5 |
| Lo Presti et al. 2019 [42] | HFpEFV | 99mTc-PYP scintigraph | 100 | unspecified ATTR-CM | 19.00% | 64% | 76 |
| Nagayoshi et al. 2023 [43] | HF Patients | Endomyocardial biopsy and/or 99mTc‐PYP scintigraphy | 50 | wtATTR-CM | 20% | 74% | 79.1 |
| Peters et al. 2022 [44] | Hospital admitted for HF | wtATTR-CM ICD9/10 codes | 205,545 | wtATTR-CM | 0.31% | - | 81 years (median) |
| Rocio Ruiz Hueso et al. 2021 [45] | HF with LVH in ≥65 years of age | 99mTc-DPD scintigraphy + transthyretin gene sequencing | 453 | unspecified ATTR-CM | 17% | 60.4% | - |
|  |  |  |  | ATTRv-CM | 5.20% |  |  |

Abbreviations: ATTR-CM = amyloid transthyretin cardiomyopathy; ATTRv-CM = amyloid transthyretin with variant cardiomyopathy; EHR: Electronic Health Records; HF = heart failure; HFrEF = Heart Failure with Reduced Ejection Fraction; HFpEF = Heart Failure with Preserved Ejection Fraction; ICD-9/10 = International Classification of Diseases, Ninth/Tenth Revision; LVH = Left Ventricular Hypertrophy; LVEF = Left Ventricular Ejection Fraction; NLP = Natural Language Processing; NYHA = New York Heart Association; PYP = pyrophosphate; SPECT = single photon emission computed tomography; Tc-DPD = technetium-99m 3,3-diphosphono-1,2-propanodicarboxylic acid; TTR = transthyretin; wtATTR-CM = wild-type amyloid transthyretin cardiomyopathy

# **Table S5**: Clinical Studies Reporting the Prevalence of ATTR in Patients with various High-risk Conditions.

| **Study** | **Study Population** | **Case Identification Method** | **Sample Size** | **ATTR type (phenotype/genotype)** | **Proportion, (%)** | **Males (%)** | **Mean Age (years)** |
| --- | --- | --- | --- | --- | --- | --- | --- |
| Akers et al. 2022 [57] | ≥65 years | Suspected ATTR-CM: HF+ selected conditions commonly associated with ATTR-CM Confirmed ATTR-CM**:** ICD-10 code | 183,580 | unspecified suspected ATTR-CM | 0.54% | – | 80 |
|  |  |  |  | unspecified confirmed ATTR-CM | 0.12% | – | – |
| Manning et al. 2020 [58] | Elderly males (≥65 years of age | HDP | 1,530 | unspecified ATTR-CM | 0.85% | 100% | 83 |
| Aimo et al. 2021 [19] | History of (CTS, LSS, etc.)/echocardiographic red flags/hs-troponin T higher than the upper reference limit in 65+ years of age | Tc-DPD | 266 | wtATTR-CM | 0.80% | - | - |
| Di Stefano et al. 2022 [57] | Patients with a sensory-motor idiopathic PN and 2+ red-flag (e.g., Family history of PN or CM, CTS, etc.) | Genetic screening | 235 | ATTRv-PN | 17.00% | - | - |
| Fukuzawa et al. 2020 [47] | Severe AS requiring surgery | Tc-99m PYP | 44 | unspecified ATTR-CM | 9.00% | 75% | >70 years |
| Gallone et al. 2022 [48] | AS undergoing TAVR evaluation | 99mTc–DPD | 107 | unspecified ATTR-CM | 7.10% | - | - |
| Gannon et al. 2023 [46] | CTS | Biopsy | 156 underwent CTR, of whom 62 got biopsy | ATTR-CM | 22.50% | 71.4% | 69.6 |
| Goena et al. 2022 [52] | Suspected ATTR based on ESC recommended criteria (IVS >12mm and a red flag) | 99mTc-DPD | 229 | unspecified ATTR-CM | 48.00% | 75.5% | 77.3 |
| Grande-Trillo et al. 2020 [56] | patients who received a DLT from patients with ATTR amyloidosis | Scintigraphy | 23 | ATTRv-CM | 4.30% | - | - |
| Morton et al. 2022 [49] | Patients undergoing TAVR | Scintigraphy | 139 | unspecified ATTR-CM | 4.32% | - | - |
| Muto et al. 2020 [50] | AS screened for ATTR | tc-99mPYP and biopsy | 32 | unspecified ATTR-CM | 18.80% | - | - |
| Nitsche et al. 2020 [5] | AS scheduled for TAVR | CMR/99mTc-DPD | 238 | unspecified ATTR-CM | 6.30% | - | - |
| Nitsche et al. 2021 [6] | AS referred for TAVR | Scintigraphy and biopsy | 407 | unspecified ATTR-CM | 11.50% | 49.8% | 83.4 |
| Prasad et al. 2022 [53] | AF + LVH in >60 years of age | Tc-99mPYP SPECT/CT | 95 | unspecified ATTR-CM | 2.00% | - | - |
| Saito et al. 2021 [54] | LVH in elderly | Biopsy or 99 mTc-PYP | 319 | unspecified ATTR-CM | 14.10% | - | - |
| Singal et al. 2021 [51] | Older than 65 years of age with severe AS being planned for SAVR | 99m-technetium PYP and biopsy | 46 | unspecified ATTR-CM | 68.00% | 70% | 70 |
| Vianello et al. 2021 [46] | 1689 CTS | electrocardiography/echocardiography/and scintigraphy | 1689 | unspecified ATTR-CM in entire study population | 4.00% | 100% | - |
|  |  |  |  | unspecified ATTR-CM in bilateral CTsS+LVH | 33.00% |  |  |

Abbreviations: AF = Atrial Fibrillation; AS = Aortic Stenosis; ATTR-CM = Transthyretin Amyloid Cardiomyopathy; ATTRv-CM = amyloid transthyretin with variant cardiomyopathy; ATTRv-PN = Variant Transthyretin Polyneuropathy; CTS = Carpal Tunnel Syndrome; CMR = Cardiac Magnetic Resonance; CTR = Carpal Tunnel Release; hs-troponin T = High-sensitivity Troponin T; IVS = Interventricular Septum; LSS = Lumbar Spinal Stenosis; LVH = Left Ventricular Hypertrophy; SAVR = Surgical Aortic Valve Replacement; SPECT/CT = Single Photon Emission Computed Tomography/Computed Tomography; TAVR = Transcatheter Aortic Valve Replacement; Tc-99m PYP = Technetium-99m Pyrophosphate; Tc-DPD = Technetium-labeled Diphosphono-Propanodicarboxylic Acid; wtATTR-CM = Wild-Type Transthyretin Amyloid Cardiomyopathy.

# **Table S6:** Clinical studies on all-cause mortality rates of among patients with ATTR: detailed exposition across all study groups and/or conditions

| **Author** | **Year** | **Country** | **Subgroup** | **Size (*n*)** | **Median Follow–up (months)** | **Median Survival (months)** | **Mortality risk** | | | | **Age (years)** | **Male Sex** |
| --- | --- | --- | --- | --- | --- | --- | --- | --- | --- | --- | --- | --- |
|  |  |  |  |  |  |  | **1 year** | **2 years** | **5 years** | **Overall** |  |  |
| Ali et al. 2020 [96] | 2020 | USA | ATTR | 357 | 20.4 | – | – | 47% | – | – | 76 | – |
|  |  |  | ATTR with AS | – | – | – | – | 33% | – | – | – | – |
| Amadio et al. 2022 [97] | 2022 | USA | ATTRv | 170 | – | 59.9 | – | – | – | – | – | – |
|  |  |  | Overall wtATTR-CM, ATTRv-CM | 2539 | – | – | – | – | – | – | 67 | 71% |
|  |  |  | wtATTR | 530 | – | 50 | – | – | – | – | – | – |
| Amaka et al. 2022 [81] | 2022 | USA | wtATTR overall | 108 | 60 | – | – | – | 49% | – | – | – |
| Arana et al. 2022 [98] | 2022 | Spain | ATTR | 266 | 13 | – | 50% | – | – | – | 77.7 | 90% |
| Bandera et al. 2022 [99] | 2022 | UK | ATTR - no SR | 342 | – | 51 | 2% | 28% | 70% | – | 76.9 | 92% |
|  |  |  | ATTR - SR with LAMC | 439 | – | 58 | 1% | 23% | 50% | – | 74 | 80% |
|  |  |  | ATTR - SR without LAMC | 125 | – | 44 | 2% | 26% | 62% | – | 75.3 | 83% |
| [Bézard et al.](https://onlinelibrary.wiley.com/doi/full/10.1002/ejhf.2589#ejhf2589-bib-0002) 2021 [s1] | 2021 | France | ATTR (on tafamidis) | 97 | – | – | 0% | 21% | 50% | – | 69 | 74% |
|  |  |  | ATTR (SC) | 531 | – | – | 13% | 37% | 80% | – | 78 | 83% |
|  |  |  | ATTRv | 205 | – | – | 14% | 21% | 58% | – | 76 | 81% |
|  |  |  | ATTRv (neuropathy only) | 16 | – | – | 0% | 17% |  | – | 76 | 81% |
|  |  |  | wtATTR | 422 | 52 | – | 18% | 27% | 71% | – | 70 | 85% |
| Bhattacharya et al. 2022 [100] | 2022 | USA | ATTR | 200 | 180 | – | – | – | – | 65% | – | – |
| Bukhari et al. 2020 [101] | 2020 | USA | ATTR | 124 | 18 | – | – | – | – | 22% | 87 | 80% |
| Bustillo et al. 2020 [s2] | 2020 | Spain | ATTR | 44 | 14 | – | – | – | – | 14% | 83 | 77% |
| [Cappelli et al.](https://onlinelibrary.wiley.com/doi/full/10.1002/ejhf.2589#ejhf2589-bib-0090) 2020 [s3] | 2020 | Italy | (by H/CL ratio) | – | – | – | – | – | – | – | – | – |
|  |  |  | ATTR stage I | 69 | 64 | – | 5% | 26% | 67% | – | – | – |
|  |  |  | ATTR stage II | 53 | 44 | – | 9% | 61% | – | – | – | – |
|  |  |  | ATTR stage III | 53 | 20 | – | 56% | – | – | – | – | – |
| Chacko et al. 2020 [103] | 2020 | UK | ATTRv - other | 33 | – | – | – | – | – | – | 58.6 | 82% |
|  |  |  | ATTRv - T60A | 127 | >60 | – | – | 22% | – | 34% | 65.6 | 68% |
|  |  |  | ATTRv - VI22I | 314 | 36 | – | – | 34% | – | 50% | 77.5 | 73% |
|  |  |  | Overall wtATTR-CM, ATTRv-CM | 1240 | – | – | – | 23% | – | 39% | – | – |
|  |  |  | wtATTR | 766 | 58 | – | – | 19% | – | 37% | 77.5 | 95% |
| Chandrashekar et al. 2020 [s4] | 2020 | USA | Tafamidis treated ATTR-CM | 51 | 8 | – | – | – | – | 16% | 73 | 100% |
| [Cheng et al.](https://onlinelibrary.wiley.com/doi/full/10.1002/ejhf.2589#ejhf2589-bib-0094) 2020 [s6] | 2020 | USA | ATTR - high-dose diuretics | 44 | – | – | 38% | 72% | 92% | – | – | – |
|  |  |  | ATTR - low-dose diuretics | 139 | – | – | 10% | 25% | 72% | – | – | – |
|  |  |  | ATTR - mid-dose diuretics | 109 | – | – | 0% | 8% | 36% | – | – | – |
|  |  |  | ATTR - no diuretics | 309 | 23 | – | 13% | – | 63% | – | 73.2 | 84% |
| Choi et al. 2020 [s6] | 2020 | Australia | Continued diflunisal for >6 months | 31 | 60 | 41 | – | – | – | – | – | – |
|  |  |  | Discontinued diflunisal within 6 months | 30 | 60 | 53 | – | – | – | – | – | – |
| Cuscaden et al. 2020 [s7] | 2020 | Australia | underwent HMDP or MDP | 6918 | 24 | – | – | – | – | – | – | – |
| [Dale et al.](https://onlinelibrary.wiley.com/doi/full/10.1002/ejhf.2589#ejhf2589-bib-0099) 2022 [s8] | 2022 | USA | ATTR - ICD + tafamidis | 18 | – | – | 0% | 0% | 40% | – | – | – |
|  |  |  | ATTR - no ICD + tafamidis | 65 | 21.1 | – | 5% | 17% | 47% | – | 73.5 | 94% |
| [Dalia et al.](https://onlinelibrary.wiley.com/doi/full/10.1002/ejhf.2589#ejhf2589-bib-0100) 2022 [104] | 2022 | USA | wtATTR - exercise duration >5.5 min | 11 | 12 | – | 12% | – | – | – | 82 | 82% |
|  |  |  | wtATTR - Exercise duration ≤5.5 min | 22 | 12 | – | 33% | – | – | – | 82 | 77% |
| Damy et al. 2021 [29] | 2021 | France | ATTR | 4815 | 84 | 33.7 | 31% | 50% | – | – | 80 | 67% |
| Donnellan et al. 2020a [105] | 2020 | USA | ATTR Gillmore stage 1 | 60 | – | – | – | – | – | 16% | – | – |
|  |  |  | ATTR Gillmore stage 2 | 105 | – | – | – | – | – | 64% | – | – |
|  |  |  | ATTR Gillmore stage 3 | 100 | – | – | – | – | – | 89% | – | – |
|  |  |  | ATTR with AF | 265 | – | – | – | – | – | 65% | 78 | 85% |
|  |  |  | ATTR without AF | 117 | – | – | – | – | – | 49% | 73 | 85% |
|  |  |  | ATTR-CM | 382 | 35 | – | – | – | – | 60% | – | – |
|  |  |  | ATTRv subgroup | 111 | – | – | – | – | – | 58% | – | – |
|  |  |  | Total wtATTR-CM, ATTRv-CM patients | – | – | – | – | – | – | 60% | – | – |
|  |  |  | wtATTR subgroup | 271 | – | – | – | – | – | 61% | – | – |
| Donnellan et al. 2020b [106] | 2019 | USA | ATTR - AF ablation | 24 | – | – | 4% | 22% | – | – | 74 | 96% |
|  |  |  | ATTR - no AF ablation | 48 | – | – | 25% | 32% | – | – | – | – |
| [Driggin et al.](https://onlinelibrary.wiley.com/doi/full/10.1002/ejhf.2589#ejhf2589-bib-0106) 2020 [107] | 2020 | USA | ATTR - mBMI high | 73 | – | – | 3% | 11% | 32% | – | – | – |
|  |  |  | ATTR - mBMI low | 182 | – | – | 10% | 24% | 58% | – | 76 | 86% |
| Fattouh et al. 2021 [s9] | 2021 | USA | wtATTR | 136 | 48 | – | – | – | – | 43% | 77 | 59% |
| Gawor et al. 2022 [s10] | 2022 | Poland | ATTRv | 10 | 20 | – | – | – | – | 10% | 58 | 100% |
| Ghoneem et al. 2022 [s11] | 2022 | USA | Non-Tafamidis^a^ | 421 | 12 | – | 16% | – | – | – | 76.2 | 86% |
|  |  |  | Tafamidis^a^ treated | 421 | 12 | – | 10% | – | – | – | 76.8 | 85% |
| Gonzalez-Lopez et al. 2022 [108] | 2022 | Spain, Italy, France, Finland, and US | ATTRv/ wtATTR-CM | 118 | 44.4 | – | 5% | – | 18% | – | 66 | 78% |
| Hein et al. 2021 [109] | 2021 | Germany | ATTR | 89 | 14.8 | – | – | – | – | 10% | – | – |
| Hoerbrand et al. 2023 [110] | 2023 | Germany | ATTR patients with Tricuspid regurgitation undergoing TTVR | – | 3 | – | – | – | – | 0% | 80 | 75% |
| Hussain et al. 2022a [111] | 2022 | USA | ATTR-CM positive (Total) | 580 | ~36 | – | – | – | – | 53% | 74 | 69% |
|  |  |  | ATTR-CM positive with AU | – | – | – | – | – | – | 53% | – | – |
|  |  |  | ATTR-CM positive without AU | – | – | – | – | – | – | 44% | – | – |
|  |  |  | ATTR-CM with AF | – | – | – | – | – | – | 60% | – | – |
|  |  |  | ATTR-CM with AF and AU | – | – | – | – | – | – | 56% | – | – |
|  |  |  | ATTR-CM with AF and without AU | – | – | – | – | – | – | 50% | – | – |
| Hussain et al. 2022b [s12] | 2022 | USA | NHYA II, non Tafamidis | 3 | – | 12.1 | – | – | – | – | – | – |
|  |  |  | NHYA II, Tafamidis | 20 | – | 80.4 | – | – | – | – | – | – |
|  |  |  | NHYA III, non Tafamidis | 23 | – | 32 | – | – | – | – | – | – |
|  |  |  | NHYA III, Tafamidis | 30 | – | 71 | – | – | – | – | – | – |
|  |  |  | non Tafamidis | 44 | – | 17.2 | – | – | – | – | 84 | 72% |
|  |  |  | suspected ATTR | 107 | 13.5 | – | – | – | – | – | 84 | 79% |
|  |  |  | Tafamidis | 63 | – | 80.4 | – | – | – | – | 83 | 84% |
| Jang et al. 2022 [112] | 2022 | South Korea | ATTR-CM | 715 | 132 | 18 | – | – | – | 35% | 69.3 | 62% |
|  |  |  | ATTR-CM < 65 years | 48 | 144 | – | – | – | – | 19% | – | – |
|  |  |  | ATTR-CM ≥ 65 years | 127 | 156 | – | – | – | – | 42% | – | – |
|  |  |  | Female ATTR-CM | 65 | 180 | – | – | – | – | 37% | – | – |
|  |  |  | Male ATTR-CM | 110 | 168 | – | – | – | – | 35% | – | – |
| Lauppe et al. 2022 [26] | 2022 | Sweden | ATTR-CM | 1930 | 132 | 30 | – | – | – | – | 73 | 69% |
|  |  |  | Female ATTR-CM patients | 598 | – | 22 | – | – | – | – | 73 | – |
|  |  |  | Male ATTR-CM patients | 1332 | – | 36 | – | – | – | – | 73 | – |
|  |  |  | Matched HF patients | 1922 | – | 67 | – | – | – | – | – | – |
| Lauppe et al. 2021 [27] | 2021 | Sweden | ATTR | 994 | – | – | 24% | 38% | 64% | – | 73 | 70% |
| [Law et al. 2020](https://onlinelibrary.wiley.com/doi/full/10.1002/ejhf.2589#ejhf2589-bib-0127) [s13] | 2020 | UK | ATTRv (V122I) | 218 | – | – | – | 52% | – | – | 77 | 71% |
|  |  |  | wtATTR | 727 | – | – | – | 31% | – | – | 79 | 94% |
| [Law et al. 2022](https://onlinelibrary.wiley.com/doi/full/10.1002/ejhf.2589#ejhf2589-bib-0128) [s14] | 2021 | UK | wtATTR (NT-proBNP <500 ng/ml) | 239 | 12 | – | 5% | 38% | 70% | – | – | – |
|  |  |  | wtATTR (NT-proBNP >500 ng/ml) | 193 | 12 | – | 1% | 25% | 65% | – | 77 | 95% |
| Leon Cejas et al. 2020 [113] | 2020 | Argentina | ATTR-PN | 94 | – | – | – | – | – | 13% | 35 | 52% |
| Longinow et al. 2023 [s15] | 2023 | USA | ATTR CM and AL CA without PH71 | 99 | 60 | – | – | – | – | 78% | – | – |
|  |  |  | ATTR CM and AL CM with PH71 | 33 | 60 | – | – | – | – | 71% | – | – |
| Milani et al. 2020 [s16] | 2020 | Italy | Overall wtATTR-CM | 229 | 21 | – | – | – | – | 17% | 76 | – |
| [Mitrani et al.](https://onlinelibrary.wiley.com/doi/full/10.1002/ejhf.2589#ejhf2589-bib-0131) 2021 [s17] | 2021 | USA | ATTR - NOACs | 116 | – | – | 10% | 28% | 80% | – | – | – |
|  |  |  | ATTR - warfarin | 78 | 28.8 | – | 10% | 24% | 78% | – | 75.2 | 89% |
| Nakashima et al. 2022 [s18] | 2022 | Japan | Overall wtATTR-CM | 188 | 21 | 59.7 | – | – | – | 25% | 78 | 85% |
| Nakov et al. 2019 [s19] | 2019 | Bulgaria | ATTRv with Glu89Gln mutation | 78 | ~33 | – | – | – | – | 28% | 57 | 51% |
| Nativi-Nicolau et al. 2020 [114] | 2020 | USA | ATTR-CM | 894 | 6 | – | – | – | – | 22% | – | 63% |
|  |  |  | HF patients | 894 | 6 | – | – | – | – | 13% | – | 46% |
| [Nitsche et al.](https://onlinelibrary.wiley.com/doi/full/10.1002/ejhf.2589#ejhf2589-bib-0010) 2020 [5] | 2020 | Austria | ATTR | 15 | – | – | 20% | – | – | – | 84 | 63% |
| [Oghina et al.](https://onlinelibrary.wiley.com/doi/full/10.1002/ejhf.2589#ejhf2589-bib-0137) 2021 [91] | 2021 | France | ATTR – NT-proBNP increasing | 121 | – | – | 58% | 80% | – | – | – | – |
|  |  |  | ATTR – NT-proBNP stable | 333 | 14.2 | – | 41% | 62% | – | – | 77 | 82% |
| Patel et al. 2022 [s20] | 2022 | UK | Non Tafamidis | 64 | 24 | ~20 | – | 35% | – | – | – | – |
|  |  |  | Tafamidis | 47 | 24 | ~20.5 | – | 17% | – | – | – | – |
| Rashdan et al. 2020 [115] | 2020 | USA | ATTR-CM + LVEF <50% | 37 | – | – | – | – | – | 35% | – | – |
|  |  |  | ATTR-CM + LVEF ≥50% | 53 | – | – | – | – | – | 23% | – | – |
|  |  |  | Total ATTR-CM patients | 91 | ~28 | – | – | – | – | 28% | 73 | 92% |
| Righetto et al. 2021 [116] | 2021 | Italy | ATTR | 67 | – | – | – | – | – | 60% | – | 75% |
| [Rosenblum et al.](https://onlinelibrary.wiley.com/doi/full/10.1002/ejhf.2589#ejhf2589-bib-0013) 2021 [s21] | 2021 | USA | ATTR on diflunisal | 13 | – | – | 7% | 7% | 63% | – | – | – |
|  |  |  | ATTR on SC | 91 | 25 | – | 20% | 40% | 87% | – | 75 | 85% |
|  |  |  | ATTR on Tafamidis | 16 | 25 | – | 6% | 12% | 37% | – | 75 | 97% |
| Salvalaggio et al. 2022 [117] | 2022 | Italy | ATTR | 67 | 47 | – | – | – | – | 61% | 78 | 65% |
| Sarkar et al. 2022 [s22] | 2022 | USA | ≥ 80 years not on Tafamidis | 59 | 18 | 18.8 | – | – | – | 80% | – | – |
|  |  |  | ≥ 80 years on Tafamidis | 110 | 18 | 38.1 | – | – | – | 60% | – | – |
|  |  |  | Gillmore stage 1, ≥ 80 years not on Tafamidis | 10 | 18 | – | – | – | – | 47% | – | – |
|  |  |  | Gillmore stage 1, ≥ 80 years on Tafamidis | 43 | 18 | – | – | – | – | 25% | – | – |
| [Shimoni et al.](https://onlinelibrary.wiley.com/doi/full/10.1002/ejhf.2589#ejhf2589-bib-0052)2021 [118] | 2021 | Israel | ATTR | 11 | 38 | – | 13% | 13% | – | – | 81.7 | 7% |
| Siddiqi et al. 2022 [s23] | 2022 | USA | Control group | 69 | 40.8 | – | – | – | – | 70% | 77 | 97% |
|  |  |  | Diflunisal group | 35 | 40.8 | – | – | – | – | 11% | 74 | 97% |
| Siegismund et al. 2018 [s24] | 2018 | Germany | ATTR with intramyocardial inflammation | 10 | – | – | – | – | – | 20% | 74 | 90% |
|  |  |  | ATTR without intramyocardial inflammation | 16 | – | – | – | – | – | 14% | 70 | 69% |
|  |  |  | Overall ATTR-CM, AL-CA | 54 | 36 | – | – | – | – | 30% | – | - |
| Silverii et al. 2022 [93] | 2022 | Italy | overall wtATTR-CM, ATTRv | 75 | ~25 | – | 15% | – | – | 16% | 80 | 91% |
| Slama et al. 2020 [119] | 2020 | France | ATTR-CM | 4815 | 24 | 33.7 | 28% | – | – | 36% | 78 | 67% |
| Sperry et al. 2018 [120] | 2018 | USA | overall wtATTR-CM, ATTRv | 54 | 21.48 | – | – | – | – | 48% | 78 | 76% |
| [Ungerer et al.](https://onlinelibrary.wiley.com/doi/full/10.1002/ejhf.2589#ejhf2589-bib-0071) 2021 [s25] | 2021 | Germany | ATTRv - NT-proBNP <900 pg/ml | 72 | – | – | 6% | 12% | 30% | – | – | – |
|  |  |  | ATTRv - NT-proBNP >900 pg/ml | 80 | 55.2 | – | 23% | 30% | 59% | – | 60.8 | – |
| Uusitalo et al. 2022 [121] | 2022 | Finland | ATTR | 17 | 12 | – | – | – | – | 88% | 80 | 78% |
| [Vaidya et al.](https://onlinelibrary.wiley.com/doi/full/10.1002/ejhf.2589#ejhf2589-bib-0155) 2021 [s26] | 2021 | USA | ATTRv - undergoing CLT | 18 | – | – | 8% | 10% | – | – | 66.8 | – |
|  |  |  | wtATTR - undergoing CLT | 18 | – | – | 7% | 10% | – | – | 71.2 | – |
| Vranian et al. 2018 [122] | 2018 | USA | ATTR | 48 | – | – | – | – | – | 56% | 78 | 73% |
| Wang et al. 2022 [s27] | 2022 | China | ATTRv-CM | 29 | 60 | 47.6 | 9% | – | 62% | – | 53 | 93% |
| [Withers et al.](https://onlinelibrary.wiley.com/doi/full/10.1002/ejhf.2589#ejhf2589-bib-0156) 2022 [s28] | 2022 | Australia | wtATTR | 12 | 63 | – | 0 | 0.15 | 0.56 | – | 76 | 92% |
| [Yamada et al.](https://onlinelibrary.wiley.com/doi/full/10.1002/ejhf.2589#ejhf2589-bib-0157) 2020 [s29] | 2020 | Japan | wtATTR - on tafamidis | 129 | 15 | – | 0.1 | 0.3 | 0.52 | – | 78.5 | 85% |
| Zareh et al. 2020 [s30] | 2020 | USA | Non amyloid HF | 84 | 45 | – | – | – | – | 0.22 | 68 | 57% |
|  |  |  | pV142I ATTRv-CM | 30 | 45 | – | – | – | – | 0.57 | 71 | 67% |
| Zhang et al. 2020 [123] | 2020 | USA | ATTR | 136 | 24 | – | – | – | – | 0.49 | – | – |

Abbreviations: AF = atrial fibrillation; AL = amyloid light-chain; AS = aortic stenosis; ATTR = transthyretin amyloidosis; ATTR-CM = transthyretin amyloid cardiomyopathy; AU = atrial uptake; CLT = cardiac light-chain transthyretin; ECG = electrocardiogram; HF = heart failure; HFpEF = heart failure with preserved ejection fraction; HFrEF = heart failure with reduced ejection fraction; ATTRv = amyloid transthyretin with variant; ICD = implantable cardioverter defibrillator; LAMC = left atrial myocardial contraction; LVH = left ventricular hypertrophy; LVEF = left ventricular ejection fraction; mBMI = modified body mass index; MDP = methyl diphosphonate; NOACs = novel oral anticoagulants; NT-proBNP = N-terminal pro b-type natriuretic peptide; NYHA = New York Heart Association (classification); PH = pulmonary hypertension; PYP = pyrophosphate; SC = standard care; SR = sinus rhythm; TTVR = transcatheter tricuspid valve repair; wtATTR = wild-type transthyretin amyloidosis
^a^Tafamdis is probably a misspelling of tafamidis, a drug for the treatment of ATTR.

# **References:**

1. Bézard M, Kharoubi M, Galat A, et al. Natural history and impact of treatment with tafamidis on major cardiovascular outcome-free survival time in a cohort of patients with transthyretin amyloidosis. Eur J Heart Fail. 2021 Feb;23(2):264-274.
2. Bustillo SR, Coca JMM, More SV, et al. Transthyretin cardiac amyloidosis: patient characteristics in our region. J Card Fail. 2020;26(10):S163.
3. Cappelli F, Martone R, Gabriele M, et al. Biomarkers and prediction of prognosis in transthyretin-related cardiac amyloidosis: direct comparison of two staging systems. Can J Cardiol. 2020 Mar;36(3):424-431.
4. Chandrashekar P, Dale Z, Rashdan L, et al. Abstract 17077: Functional class, biomarker stability, and clinical outcomes of patients with transthyretin cardiac amyloidosis treated with tafamidis. Circulation. 2020;142(suppl 3):A17077.
5. Cheng RK, Levy WC, Vasbinder A, et al. Diuretic dose and NYHA functional class are independent predictors of mortality in patients with transthyretin cardiac amyloidosis. JACC CardioOncol. 2020 Sep;2(3):414-424.
6. Choi B, Lasica M, Hare J, et al. 105 Diflunisal is Effective and Affordable Treatment in Transthyretin Cardiac Amyloidosis (ATTR-CM) - but Only Half of Patients can Tolerate It. Heart, Lung and Circulation. 2020;29:S83.
7. Cuscaden C, Ramsay SC, Prasad S, et al. Estimation of prevalence of transthyretin (ATTR) cardiac amyloidosis in an Australian subpopulation using bone scans with echocardiography and clinical correlation. J Nucl Cardiol. 2021 Dec;28(6):2845-2856.
8. Dale Z, Al-Rashdan L, Elman M, et al. Mode of death and outcomes of implantable cardioverter defibrillators in transthyretin amyloid cardiomyopathy. Int J Cardiol. 2022 Feb 15;349:99-102.
9. Fattouh M, Patel A, Gilad A, et al. Abstract 13392: Impaired oxygen uptake efficiency slope (OUES) predicts mortality in wild-type transthyretin amyloid cardiomyopathy (ATTRwt-CM). Circulation. 2021;144(suppl 1).
10. Gawor M, Holcman K, Franaszczyk M, et al. Spectrum of transthyretin gene mutations and clinical characteristics of Polish patients with cardiac transthyretin amyloidosis. Cardiol J. 2022;29(6):985-993.
11. Ghoneem A, Bhatti AW, Mitchell JD, et al. Abstract 13273: Real-world efficacy of tafamidis in patients with transthyretin amyloidosis and heart failure. Circulation. 2022;146(suppl 1).
12. Hussain K, Macrinici V, Wathen L, et al. Impact of tafamidis on survival in a real-world community-based cohort. Curr Probl Cardiol. 2022 Dec;47(12):101358.
13. Law S, Petrie A, Chacko L, et al. Disease progression in cardiac transthyretin amyloidosis is indicated by serial calculation of National Amyloidosis Centre transthyretin amyloidosis stage. ESC Heart Fail. 2020 Dec;7(6):3942-3949.
14. Law S, Petrie A, Chacko L, et al. Change in N-terminal pro-B-type natriuretic peptide at 1 year predicts mortality in wild-type transthyretin amyloid cardiomyopathy. Heart. 2022 Mar;108(6):474-478.
15. Longinow J, Buggey J, Jacob M, et al. Significance of pulmonary hypertension in cardiac amyloidosis. Am J Cardiol. 2023 Apr 1;192:147-154.
16. Milani P, Cavenaghi G, Obici L, et al. Regional cardiac uptake of 99-Tc-DPD is a novel powerful and independent prognostic marker in cardiac ATTR wild type amyloidosis. Eur Heart J. 2020;41(suppl 2):2116.
17. Mitrani LR, de Los Santos J, Driggin E, et al. Anticoagulation with warfarin compared to novel oral anticoagulants for atrial fibrillation in adults with transthyretin cardiac amyloidosis: comparison of thromboembolic events and major bleeding. Amyloid. 2020;28(1):30-34.
18. Nakashima N, Takashio S, Morioka M, et al. A simple staging system using biomarkers for wild-type transthyretin amyloid cardiomyopathy in Japan. ESC Heart Fail. 2022 Jun;9(3):1731-1739.
19. Nakov R, Sarafov S, Nakov V, et al. Gastrointestinal manifestations in hereditary transthyretin amyloidosis associated with Glu89Gln mutation. J Gastrointestin Liver Dis. 2019 Dec 9;28(4):421-426.
20. Patel R, Steinberg R, Rim A, et al. Abstract 14210: Heart failure hospitalizations and mortality outcomes in PV142I transthyretin cardiomyopathy patients treated with tafamidis. Circulation. 2022;146(suppl 1):S102-S103.
21. Rosenblum H, Masri A, Narotsky DL, et al. Unveiling outcomes in coexisting severe aortic stenosis and transthyretin cardiac amyloidosis. Eur J Heart Fail. 2021 Feb;23(2):250-258.
22. Sarkar A, Miranda D, Sleiman J, et al. Does tafamidis benefit octogenarians with transthyretin amyloid cardiomyopathy? analysis of the Cleveland Clinic Amyloid Registry. J Am Coll Cardiol. 2022 2022/03/08;79(9 suppl):300.
23. Siddiqi OK, Mints YY, Berk JL, et al. Diflunisal treatment is associated with improved survival for patients with early stage wild-type transthyretin (ATTR) amyloid cardiomyopathy: the Boston University Amyloidosis Center experience. Amyloid. 2022 Jun;29(2):71-78.
24. Siegismund CS, Escher F, Lassner D, et al. Intramyocardial inflammation predicts adverse outcome in patients with cardiac AL amyloidosis. Eur J Heart Fail. 2018 Apr;20(4):751-757.
25. Ungerer MN, Hund E, Purrucker JC, et al. Real-world outcomes in non-endemic hereditary transthyretin amyloidosis with polyneuropathy: a 20-year German single-referral centre experience. Amyloid. 2021 Jun;28(2):91-99.
26. Vaidya GN, Patel JK, Kittleson M, et al. Intermediate-term outcomes of heart transplantation for cardiac amyloidosis in the current era. Clin Transplant. 2021 Jun;35(6):e14308.
27. Wang S, Peng W, Pang M, et al. Clinical profile and prognosis of hereditary transthyretin amyloid cardiomyopathy: a single-center study in South China. Front Cardiovasc Med. 2022;9:900313.
28. Withers B, McCaughan G, Hayward C, et al. Clinical characteristics and prognosis of cardiac amyloidosis defined by mass spectrometry-based proteomics in an Australian cohort. Intern Med J. 2022 Jan;52(1):69-78.
29. Yamada T, Takashio S, Arima Y, et al. Clinical characteristics and natural history of wild-type transthyretin amyloid cardiomyopathy in Japan. ESC Heart Fail. 2020 Oct;7(5):2829-2837.
30. Zareh M, Levine A, Berk JL, et al. Abstract 15242: Increased mortality among african american patients with heart failure caused by hereditary transthyretin amyloid cardiomyopathy. Circulation. 2020;142(suppl 3):A15242.
